# The Use and Perception of Electronic Cigarettes and Snus among the U.S. Population 

**DOI:** 10.1371/journal.pone.0079332

**Published:** 2013-10-24

**Authors:** Shu-Hong Zhu, Anthony Gamst, Madeleine Lee, Sharon Cummins, Lu Yin, Leslie Zoref

**Affiliations:** Moores Cancer Center, University of California San Diego, La Jolla, California, United States of America; University of Alabama, United States of America

## Abstract

**Background:**

E-cigarettes have generated controversy in the tobacco control field similar to that of Swedish snus, which came to the U.S. market six years earlier. Some argue that e-cigarettes have great potential to help smokers quit regular cigarettes while others contend they should be banned for lack of safety and efficacy data. This study examined population data from the U.S.

**Methods:**

A U.S. population survey with a national probability sample (N=10,041) was conducted (February 24 to March 8, 2012, before any major paid advertisement of e-cigarettes appeared on television). Survey respondents were asked if they had heard about e-cigarettes, where they had heard about them, whether they had used e-cigarettes or snus, how often they used them, and why they used them. Responses were weighted to represent the entire U.S. population.

**Findings:**

A high proportion, 75.4%, reported having heard about e-cigarettes. Television ranked as the number one source of information, followed by “in-person conversation” and “Internet.” About 8.1% had tried e-cigarettes, and 1.4% were current users. These rates were twice those of snus (4.3% and 0.8%, respectively). Among current smokers, 32.2% had tried e-cigarettes, and 6.3% were current users. Over 80% of current e-cigarette users were non-daily users. Women were significantly more likely to have tried e-cigarettes than men. Those who had tried e-cigarettes were more likely than those who tried snus to report their products being safer than regular cigarettes (49.9% vs. 10.8%). Almost half (49.5%) of current smokers were susceptible to using e-cigarettes in the future.

**Conclusions:**

That e-cigarettes have surpassed snus in adoption rate, even before any promotion by major tobacco companies, suggests that the former have tapped into smokers’ intuitive preference for potentially harm-reducing products, probably due to the product design. E-cigarette use is likely to increase in the next few years.

## Introduction

The Electronic Nicotine Delivery System (ENDS), also known as the e-cigarette, has increasingly attracted the attention of smokers and tobacco control workers [1,2]. E-cigarettes were first developed in China in 2003 [3]. They came to the U.S. market in 2007 and quickly gained notoriety in many countries, especially those with relatively strong tobacco control programs [4-9]. Unfortunately, scientific information about e-cigarettes is limited. Some argue that e-cigarettes are obviously less harmful than cigarettes and have great potential to help smokers quit [10,11], while others contend that data on safety are needed before e-cigarettes are promoted or allowed to be sold [1]. Anecdotal reports of smokers using e-cigarettes to help them quit smoking abound [3,7,12-14], but efficacy data in the form of clinical trials are still limited [15-19].

Insufficient scientific research on the safety and efficacy of e-cigarettes is one reason that the products have attracted controversy. Some countries have banned the sale of e-cigarettes [20], although that does not prevent smokers from purchasing them on the Internet. In the U.S., the Food and Drug Administration (FDA) has attempted to regulate the sale and marketing of e-cigarettes, a move that was struck down by a federal court [21]. Short of FDA regulatory oversight, some states have tried to pass laws to ban the sale of e-cigarettes in their own jurisdictions [22] although the availability of e-cigarettes on the Internet makes it difficult to enforce such a ban. Meanwhile, the rationale for the ban itself appears to be chiefly based on predicted potential harm, as empirical evidence is sparse [11].

This controversy surrounding e-cigarettes is reminiscent of the controversy associated with another tobacco product, snus. Snus, a moist smokeless tobacco product popular in Sweden, gained the attention of global tobacco control workers a few years before e-cigarettes did [23]. Strong arguments for and against snus have been advanced, but the tobacco control field remains divided [24-27]. Especially difficult is the debate on the potential of snus to reduce the harm of tobacco use at the population level [27]. While using snus may be less risky than smoking cigarettes to the health of the individual, it is not clear that promoting the use of snus would reduce the total harm associated with tobacco use at the population level [28,29]. Some have argued that promoting the use of any tobacco product supports the tobacco-use norm and, as such, would produce a negative net-effect on tobacco control at the population level [27]. Similar difficulty exists in the current controversy on e-cigarettes.

There is, however, one noticeable difference in the short history of e-cigarettes and snus in the U.S. market. E-cigarettes seem to have achieved notoriety relatively quickly without major paid advertising [30]. Unlike snus, which has been promoted by large tobacco companies in the U.S. [31,32], e-cigarettes had not been promoted by any major tobacco company until Lorillard Inc. acquired a major brand Blu-Cigs in April 2012 [33]. Instead, e-cigarettes appear to have received much free publicity. No study has carefully documented the level of paid advertising versus earned media for e-cigarettes. But a quick web search will show that e-cigarettes have received much free coverage. For example, endorsements have come from some American celebrities and talk show hosts, who tout e-cigarettes’ intuitive appeal and how they can help smokers quit cigarettes [34,35]. E-cigarettes have appeared in popular movies [35,36]. By 2010, web searches for information on e-cigarettes in that year had surpassed those for snus in the U.S. [6] All this suggests that the adoption of e-cigarettes may be significant, and a comparison with the adoption of snus will be informative.

The present study aimed to provide some basic measures on how much of a foot-hold e-cigarettes had already taken among the U.S. population before Lorillard Inc. purchased a well-known e-cigarette brand and started a significant television advertising campaign [33,37]. Using a survey of a probability sample of the U.S. population, this study examined the knowledge about e-cigarettes among smokers and nonsmokers. It provided population prevalence measures on ever and current use of e-cigarettes and the rate of transition from ever use to current use. The perceived utility of e-cigarettes as a quitting aid or as a potential harm reduction product was assessed and compared with that of snus, a potential harm reduction product that aroused similar controversy when it came to the U.S. a few years before e-cigarettes. Finally, the proportion of the U.S. population that is susceptible to future e-cigarettes was estimated. 

## Methods

### Ethics Statement

This research was performed in accordance with a human subjects protocol approved by the University of California, San Diego’s Institutional Review Board (IRB# 111664).

### Data source

The data for this study was obtained from a survey commissioned by the University of California, San Diego and administered by Knowledge Networks (Menlo Park, CA). Knowledge Networks, which was recently acquired by GfK, recruits a probability sample representative of the U.S. population (KnowledgePanel). The sample was originally recruited by random digit dialing (RDD) but an address-based sampling methodology has been used in recent years [38]. A detailed description of the sampling methods used to recruit to the KnowledgePanel has been described elsewhere [39]. The advantages and the limitations of using the KnowledgePanel have also been discussed in many contexts and will not be repeated here [40-42]. In summary, the panel provides an efficient way of accessing a probability sample of the U.S. population, whose representativeness is similar to most other well-known population surveys [42-46]. All Knowledge Networks surveys are performed online. Knowledge Networks provides a netbook computer and network access to participants, as needed. Many health behavior studies have used the KnowledgePanel [45,47,48].

The present survey was designed to gather information on smoking history and cigarette use, perceptions about different tobacco products and quitting aids, attitudes toward tobacco control efforts, and beliefs and ideation about the process of quitting smoking. The study over sampled the smokers in the KnowledgePanel so that all the available smokers were included, with a random sub-sample of former smokers and never smokers from the panel such that the three smoking-status groups were approximately equal in size. A total of 15,095 adults (> 18 years of age) were sampled and invited to participate in the survey. Of these, 10,041 completed the survey, a response rate of 66.5%. This corresponds to a sample with 3,111 current smokers, 3,676 former smokers and 3,254 never smokers. The survey was conducted between February 24^th^ and March 8^th^, 2012.

### Measurement

Cigarette smoking behavior was assessed in multiple questions. Current smokers were defined as those who had smoked at least 100 cigarettes in their lifetime and who answered the question, “Do you currently smoke cigarettes every day, some days, or not at all?” with “every day” or “some days”. Those who had smoked at least 100 cigarettes in their lifetime and answered “not at all” were classified as former smokers. Former smokers were further asked, “When did you smoke your last cigarette?” They were categorized as recent former smokers if they selected any of the options with a time frame of 1 year or less, and long-term former smokers if they answered “Over 1 year ago”. Nonsmokers were defined as those who had not smoked 100 cigarettes in their lifetime. 

Current smokers were asked if they had ever tried to quit smoking, and if they answered yes, were also asked whether they had tried to quit in the last 12 months.

Use of snus was assessed by the question, “Have you ever used any of the following tobacco products?” for which “Snus (tobacco in a small pouch, like Camel snus or Marlboro snus)” was one of the available options. Those who selected “yes” were defined as ever users of snus and asked the question “Do you currently use snus every day, some days, or not at all?” Those selected “every day” or “some days” were defined as current users of snus. 

Use of e-cigarettes was also assessed in multiple questions. First, respondents were asked if they have ever heard of e-cigarettes: “E-cigarettes (electronic cigarettes) are electronic devices that deliver nicotine in a vapor and look like cigarettes, but contain no tobacco. Have you ever heard of e-cigarettes?” Those who had heard of e-cigarettes were also asked where they had heard about e-cigarettes and were allowed to select one or more of the following options: “Radio”, “TV”, “Internet”, “In-person conversation”, “Information shared via Facebook, YouTube, or other social network media”, and “Other”.

Additionally, those who had heard of e-cigarettes were asked: “Have you ever tried an e-cigarette”, and those who answered yes were considered ever users. Ever users were also asked “Have you used e-cigarettes in the last 30 days”, and those who answered yes were considered current users. Current users were asked to provide the number of days (in the last 30) they had used e-cigarettes.

Ever users of e-cigarettes were asked “Why did you use e-cigarettes?” and instructed to select “Yes” or “No” for each of the following options: “Safer than cigarettes”, “Cheaper than cigarettes,” “Easy to use when I can’t smoke,” “To try to quit smoking cigarettes”, or “Just because.” Since a person could have multiple reasons for using any product, the order of these options was randomized by individual respondent to minimize the order effect in response (e.g., respondent may be more likely to choose the first option on the list). The “just because” option was included to make clear that the respondent need not have any particular reason.

Finally, those who had never used e-cigarettes were asked the question: “How likely are you to try e-cigarettes in the future?” This was intended to assess their susceptibility to e-cigarettes, much like the susceptibility measure on uptake of regular cigarettes [49]. Those who responded that they were “Very likely” or “Somewhat likely” were considered to be susceptible. This is slightly stricter definition than the susceptibility measure used in the literature for uptake of cigarettes in that the present definition does not include those who responded “somewhat unlikely” [49]. The susceptibility definition here does include those who have tried e-cigarettes but are not currently using them.

### Analysis

All percentages were weighted by population parameters based on the most recent U.S. Current Population Survey [46]. A survey-specific post-stratification adjustment was used to account for any survey non-response, as well as any non-coverage or under- and over-sampling resulting from the survey-specific sampling design. In this case, this survey had approximately equal numbers of current smokers, former smokers, and never smokers. The adjustment for over-sampling of smokers produced an overall smoking prevalence for the U.S. of 19.1% based on this survey, which is quite close to the newest published national estimate of 19.3% based on the 2010 National Health Interview Survey [50]. All results were analyzed by demographic categories (gender, age, educational level, ethnic background), as well as by smoking status. Standard errors were calculated and 95% confidence intervals were computed based on the sampling distribution of the corresponding summary statistic. Confidence intervals for binomial proportions were computed using the method of Agresti and Coull ([51]; see also 52). All calculations were done using R 2.12.1 [53].

## Results

**Figure 1:**
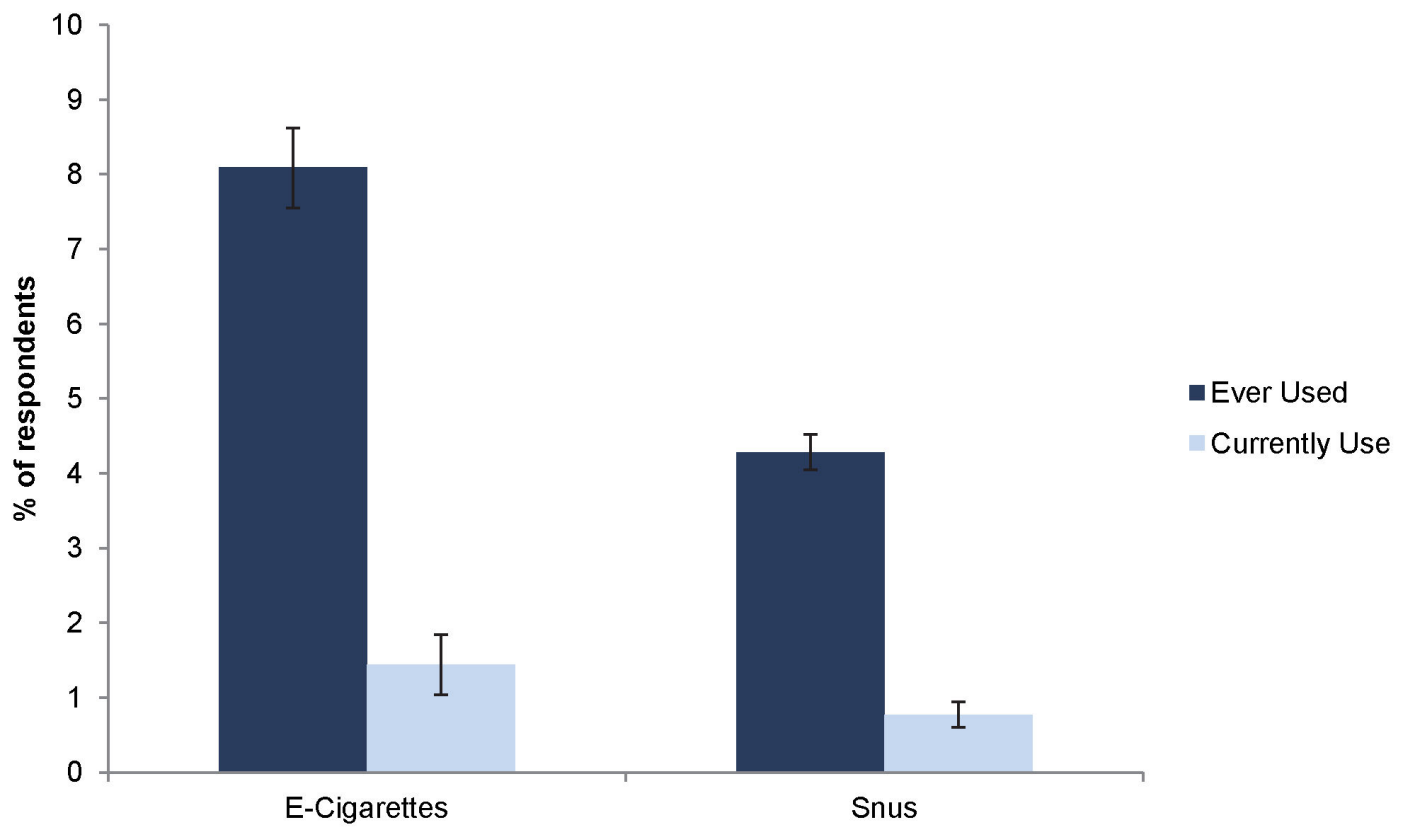
The rates of ever use and current use of E-Cigarettes and Snus.

Figure 1 shows the rate of having “ever used” and “currently use” for e-cigarettes and snus, weighted to the U.S. population. A total of 8.08% reported that they had ever used e-cigarettes, and 1.44% reported currently using e-cigarettes. Thus, approximately 18% of those who have ever used e-cigarettes continue as current users (1.44/8.08 =17.8%). 

The rate of ever used and current use for snus is approximately half that of e-cigarettes: 4.26% and 0.77%, respectively. The rate of transition from ever users of snus to current users, however, is about the same, 18% (0.77/4.26 = 18.1%).

Table 1 presents the usage rates of e-cigarettes and snus by demographic categories. It also separates out those who used only e-cigarettes or snus from those who used both products. The top half of the table shows the “ever use” rates. Women are more likely to have ever used e-cigarettes than men. The usage rate is higher among the young and those with lower education. Hispanics are less likely to have ever used e-cigarettes than either Whites or Blacks. 

**Table 1:** Ever and Current Use of E-Cigarettes and Snus (n = 10,041).

**Ever Use**				
		**E-cigarettes only**	**Snus only**	**E-Cigarettes and Snus**
		**% (95% CI)**	**% (95% CI)**	**% (95% CI)**
**Mean**		6.89 (6.40-7.39)	3.07 (2.73-3.41)	1.19 (0.98-1.40)
**Gender**	Male	5.33 (4.51-6.15)	5.26 (4.32-6.20)	1.84 (1.25-2.43)
	Female	8.33 (7.33-9.33)	1.06 (0.63-1.49)	0.59 (0.28-0.90)
**Age**	18-24	9.33 (6.59-12.07)	3.54 (1.60-5.48)	3.37 (1.49-5.25)
	25-44	7.78 (6.43-9.13)	3.98 (2.92-5.04)	1.69 (1.02-2.36)
	45-54	7.35 (6.47-8.23)	2.88 (2.23-3.53)	0.52 (0.28-0.76)
	65+	2.62 (19.3-3.31)	1.25 (0.78-1.72)	0.34 (0.00-0.69)
**Education**	≤12 years	8.60 (7.37-9.83)	3.48 (2.60-4.36)	1.61 (1.23-1.99)
	>12 years	5.63 (4.94-6.32)	2.76 (2.17-3.35)	0.88 (0.61-1.15)
**Ethnicity**	Non-Hispanic White	7.02 (6.29-7.75)	3.27 (2.66-3.88)	1.32 (0.91-1.73)
	Black	8.12 (5.55-10.69)	1.19 (0.39-1.99)	0.35 (0.00-0.74)
	Hispanic	4.38 (1.81-6.95)	4.40 (1.64-7.16)	1.42 (0.00-3.07)
	Other	6.14 (4.87-7.40)	2.94 (1.43-4.45)	1.22 (0.64-1.80)
	Multi-racial	9.51 (5.24-13.78)	4.69 (0.97-8.41)	0.52 (0.00-1.07)
**Current Use**				
		**E-cigarettes only**	**Snus only**	**E-Cigarettes and Snus**
		**% (95% CI)**	**% (95% CI)**	**% (95% CI)**
**Mean**		1.28 (1.06-1.50)	0.61 (0.46-0.76)	0.16 (0.08-0.24)
**Gender**	Male	1.00 (0.65-1.35)	0.96 (0.53-1.39)	0.22 (0.00-0.46)
	Female	1.54 (1.13-1.95)	0.29 (0.11-0.47)	0.10 (0.00-0.26)
**Age**	18-24	1.47 (0.33-2.61)	0.27 (0.00-0.58)	0.23 (0.00-0.48)
	25-44	1.19 (0.62-1.76)	0.75 (0.28-1.22)	0.29 (0.00-0.62)
	45-54	1.67 (1.34-2.00)	0.69 (0.30-1.08)	0.08 (0.00-0.22)
	65+	0.54 (0.25-0.83)	0.33 (0.02-0.64)	0.02 (0.00-0.04)
**Education**	≤12 years	1.78 (1.21-2.35)	0.81 (0.40-1.22)	0.35 (0.04-0.66)
	>12 years	0.91 (0.69-1.13)	0.47 (0.22-0.72)	0.02 (0.00-0.04)
**Ethnicity**	Non-Hispanic White	1.53 (1.18-1.88)	0.59 (0.33-0.85)	0.06 (0.00-0.14)
	Black	1.26 (0.32-2.20)	0.49 (0.00-0.99)	0.03 (0.00-0.07)
	Hispanic	0.30 (0.00-0.72)	1.87 (0.00-3.97)	0.70 (0.00-2.07)
	Other	0.58 (0.16-1.00)	0.31 (0.00-0.63)	0.54 (0.00-1.23)
	Multi-racial	0.79 (0.19-1.39)	0.16 (0.00-0.47)	0

The usage rates for snus are significantly lower. The main difference is in gender: About the same percentage of men have tried e-cigarettes or snus: 7.17 % (5.33% + 1.84%) for e-cigarettes and 7.10% (5.26% + 1.84%) for snus. However, the percentage of women having tried e-cigarettes is much higher than for snus 8.92% (8.33% + 0.59%) versus 1.65% (1.06% + 0.59%). 

The bottom half of Table 1 shows the rates of current use. The demographic pattern for the “currently use” is similar to that for “ever used” except that the rates for the former are significantly lower across all demographic categories. 

Table 2 shows the rates of ever used and current use by smoking status and by gender. In this table, all users of e-cigarettes are combined into one group regardless of whether they use snus or not. The same is done for snus users: They are combined into one group of snus users regardless of their e-cigarette use status (thus, dual users are counted in both calculations).

**Table 2:** Ever and Current Use of E-Cigarettes and Snus, by Gender and Smoking Status.

		**Never smokers (n=3,254)**	Long-term former smokers* (n=3,263)	**Recent former smokers^†^ (n=413)**	**Current smokers (n=3,111)**
		**% (95% CI)**	**% (95% CI)**	**% (95% CI)**	**% (95% CI)**
**Ever use of e-cigarettes**	Male	0.97 (0.34-1.60)	1.70 (0.92-2.48)	24.49 (16.94-32.04)	26.99 (23.07-30.91)
	Female	1.09 (0.48-1.70)	3.17 (2.05-4.29)	29.11 (21.37-36.85)	37.57 (33.55-41.59)
	Mean	1.04 (0.61-1.47)	2.40 (1.71-3.09)	26.78 (21.35-32.21)	32.18 (29.34-35.05)
**Ever use of snus**	Male	2.57 (1.39-3.75)	6.37 (4.76-7.98)	22.93 (14.66-31.20)	17.27 (13.74-20.80)
	Female	0.50 (0.21-0.79)	0.49 (0.06-0.92)	5.77 (1.77-9.77)	6.08 (3.55-8.61)
	Mean	1.43 (0.86-2.00)	3.45 (2.66-4.42)	14.43 (9.65-19.21)	11.76 (9.56-13.96)
**Current use of e-cigarettes**	Male	0.05 (0.00-0.15)	0.12 (0.00-0.26)	4.97 (1.58-8.36)	4.96 (3.07-6.87)
	Female	0.03 (0.00-0.09)	0.22 (0.00-0.47)	7.22 (2.79-11.65)	7.61 (5.41-9.81)
	Mean	0.04 (0.00-0.10)	0.17 (0.03-0.31)	6.08 (3.30-8.86)	6.26 (4.81-7.71)
**Current use of snus**	Male	0.56 (0.26-0.85)	0.64 (0.18-1.09)	2.84 (0.18-5.45)	3.20 (2.10-4.30)
	Female	0.06 (0.00-0.17)	0.23 (0.00-0.50)	0.0	1.70 (0.51-2.82)
	Mean	0.28 (0.00-0.57)	0.44 (0.11-0.77)	1.44 (0.09-2.79)	2.46 (1.46-3.46)

* Ever smokers who quit more than a year ago at the time of survey.

^†^ Ever smokers who quit within a year or less at the time of survey.

Table 2 shows that the difference in usage rates by smoking status is large. About 1% of never smokers have ever tried e-cigarettes, while over 32% of current smokers have used e-cigarettes. The same is true with ever using snus. Clearly, recent former smokers and current smokers are the most likely to have tried e-cigarette or snus. 

There is also a significant gender difference in ever using e-cigarettes or snus. This gender difference is most clearly seen among the current smokers: women are more likely to have tried e-cigarettes than men (about 38% vs. 27%). In contrast, men are more likely to have tried snus (about 17% vs. 6%). It should be noted, however, that both women and men are more likely to have tried e-cigarettes than snus.

Table 2 also shows that the current use rates have a similar pattern to the ever use rates. It is recent former smokers and current smokers who are more likely to be current users of e-cigarettes or snus. The gender difference between e-cigarette and snus use is reversed in this comparison, but the difference is not statistically significant. The overall rates, however, are significantly higher for e-cigarettes than for snus among recent formers smokers and current smokers.

Table 3 shows the frequency of use for e-cigarettes or snus among those who currently use either of these two types of products. Among e-cigarette users, there is a significant difference in the use pattern between current smokers and recent former smokers. Among current smokers, those who used e-cigarettes were mostly occasional users, only 11.5% of used e-cigarettes daily. Among recent former smokers, however, 45.7% used them on a daily basis, a statistically significant difference (p<0.05). 

**Table 3:** Frequency of Using E-Cigarettes and Snus in the Past 30 Days.

		**Never smokers**	Long-term former smokers*	**Recent former smokers^†^**	**Current smokers**	**Overall**
		**% (95% CI)**	**% (95% CI)**	**% (95% CI)**	**% (95% CI)**	
**Current use of e-cigarettes (n=267)**	**Every Day**	0	31.0 (0.0-71.7)	45.7 (20.9-70.5)	11.5 (3.3-19.7)	16.3 (8.2-24.4)
	**Some Days**	100	69.0 (28.3-100.0)	54.3 (29.5-79.1)	88.5 (80.3-96.7)	83.7 (75.6-91.8)
**Current use of snus (n=80)**	**Every Day**	11.3 (0.0-33.8)	75.3 (47.7-100.0)	15.9 (0.0-45.7)	23.2 (1.6-44.8)	27.1 (11.1-43.1)
	**Some Days**	88.7 (66.2-100.0)	24.7 (0.0-52.3)	84.1 (54.3-100.0)	76.8 (55.2-98.4)	72.9 (56.9-88.9)

* Ever smokers who quit more than a year ago at the time of survey.

^†^ Ever smokers who quit within a year or less at the time of survey.

Most snus users used snus on a non-daily basis. Among the long-term former smokers, however, most used snus on a daily basis. 

Table 4 shows the reasons of use, reported by the ever e-cigarette users and ever snus users. It is useful to take a look at those who used both products, first (the last two columns of the table). The most common reason for having tried e-cigarettes or snus is “just because:” 72.3% for e-cigarettes and 82.1% for snus. For e-cigarettes, the second most common reason given is “to try to quit smoking cigarettes,” followed by “safer than cigarettes” and “easy to use when I can’t smoke.” For snus, the second most common reason is “easy to use when I can’t smoke,” “followed by to try to quit smoking cigarettes.” Overall, the dual users are significantly more likely to report use of e-cigarettes than snus to quit smoking, 56.9% vs. 30.1%. Dual users are more likely to report the belief that e-cigarettes are safer than cigarettes, 58.2% vs. 26.2% for snus. 

**Table 4:** Reasons for Having Tried E-Cigarettes and Snus*.

	**Used either e-cigarettes or snus**		**Used both e-cigarettes and snus**	
	**E-Cigarettes (n=1,057)**	**Snus (n=316)**	**E-Cigarettes (n=122)**	**Snus (n=122)**
**Safer than cigarettes**	49.9 (44.7-55.1)	10.8 (5.9-15.7)	58.2 (44.7-71.7)	26.2 (13.8-38.5)
**Cheaper than cigarettes**	30.3 (25.7-34.9)	24.6 (16.7-32.5)	36.9 (22.8-51.0)	24.8 (13.6-36.0)
**Easy to use when I can't smoke**	44.8 (39.7-49.9)	37.6 (29.2-46.0)	57.4 (43.7-71.1)	49.9 (36.0-63.8)
**To try to quit smoking cigarettes**	54.9 (49.8-60.0)	26.3 (19.0-33.6)	56.9 (43.3-70.5)	30.1 (17.7-42.5)
**Just because**	68.3 (63.8-72.8)	73.8 (66.6-81.0)	72.3 (57.7-85.9)	82.1 (73.3-90.9)

* The order of these options was randomized for individual respondents to minimize the order effect in response.

The patterns for those using either e-cigarettes only or snus only are presented in columns 1 and 2, and they are similar to that of dual users. E-cigarette users are about twice as likely as snus users to report using the product “to try to quit smoking” (54.9% vs. 26.3%). The former are also significantly more likely to believe e-cigarettes are safer than regular cigarettes than the latter are to believe snus is safer than cigarettes (49.9% vs. 10.8%).

**Figure 2:**
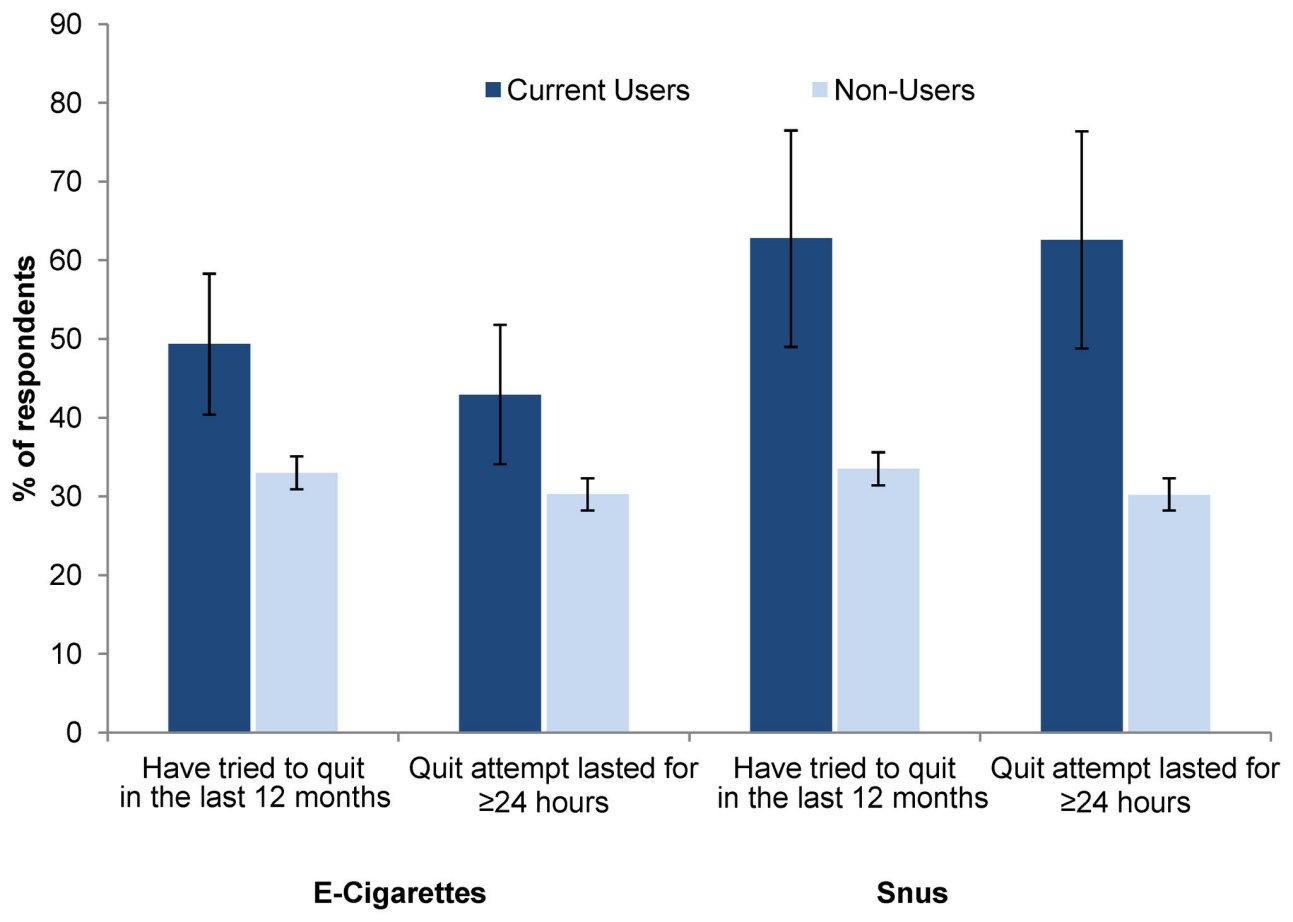
Quit attempts among current users of E-Cigarettes and Snus versus non-users.

Figure 2 shows that those who are currently using e-cigarettes are significantly more likely to have tried to quit smoking in the last 12 months than those who are currently not using e-cigarettes. The former are also more likely to have made an attempt that lasted for at least 24 hours (both p’s < 0.05). The same is true for snus use: those who are currently using snus are more likely to have tried to quit smoking than those not currently using snus. The former are also more likely to have made an attempt that lasted for at least 24 hours (both p’s < 0.05). 

Table 5 shows data on those reporting having “ever heard of e-cigarettes” and where they heard about them. Three quarters of survey respondents, 75.4%, reported that they have heard of e-cigarettes. The rate of awareness is high across gender, age, education level and ethnicity. Even 69.2% of never smokers have heard about e-cigarettes, and the percentage goes up to 88.1% for current smokers. 

**Table 5:** Awareness of E-Cigarettes.

		**Ever heard of e-cigarettes (n=8,045)**	**Heard of on radio (n=1,019)**	**Heard of on television (n=3,806)**	**Heard of on the internet (n=1,850)**	**Heard of in-person (n=3,178)**	**Heard of on social networks (n=199)**	**None of the above (n=1,473)**
		**% (95% CI)**	**% (95% CI)**	**% (95% CI)**	**% (95% CI)**	**% (95% CI)**	**% (95% CI)**	**% (95% CI)**
	**Average**	75.4 (74.1-76.7)	12.2 (11.2-13.2)	48.0 (46.4-49.6)	20.7 (19.4-22.0)	38.2 (36.3-39.8)	2.7 (2.1-3.3)	18.0 (16.7-19.3)
**Gender**	Male	78.9 (77.1-80.7)	13.8 (12.2-15.4)	49.4 (47.0-51.8)	25.2 (23.2-27.2)	34.2 (32.0-36.4)	2.9 (2.1-3.7)	17.2 (15.4-19.0)
	Female	72.3 (70.3-74.3)	10.5 (9.1-11.9)	46.6 (44.2-49.0)	16.3 (14.5-18.1)	42.1 (39.7-44.5)	2.6 (1.8-3.4)	18.7 (16.9-20.5)
**Age**	18-24	76.7 (71.8-81.6)	8.9 (5.4-12.4)	41.6 (35.1-48.1)	25.1 (19.4-30.8)	46.0 (39.3-52.7)	3.2 (1.2-5.2)	14.7 (10.4-19.0)
	25-44	74.9 (72.4-77.4)	13.2 (11.2-15.2)	40.7 (37.6-43.8)	23.6 (21.1-26.1)	43.5 (40.4-46.6)	3.8 (2.6-5.0)	19.3 (16.8-21.8)
	45-64	78.0 (76.2-79.8)	12.3 (10.9-13.7)	52.7 (50.3-55.1)	19.5 (17.7-21.3)	36.0 (33.8-38.2)	1.8 (1.2-2.4)	16.9 (15.1-18.7)
	65+	70.2 (67.7-72.7)	11.3 (9.1-13.5)	57.3 (54.2-60.4)	14.4 (12.4-16.4)	26.2 (23.5-28.9)	2.2 (0.8-3.6)	19.3 (16.8-21.8)
**Education**	≤12 years	73.1 (70.9-75.3)	11.1 (9.5-12.7)	51.8 (49.1-54.5)	17.3 (15.3-19.3)	37.0 (34.5-39.5)	2.7 (1.7-3.7)	15.6 (13.6-17.6)
	>12 years	77.2 (75.4-79.0)	12.9 (11.5-14.3)	45.3 (43.1-47.5)	23.2 (21.4-25.0)	39.0 (37.0-41.0)	2.7 (1.9-3.5)	19.6 (17.8-21.4)
**Ethnicity**	Non-Hispanic White	80.6 (79.4-81.8)	12.4 (11.2-13.6)	47.4 (45.6-49.2)	20.2 (18.8-21.6)	38.5 (36.7-40.3)	2.6 (2.0-3.2)	17.4 (16.0-18.8)
	Black	62.3 (57.4-67.2)	11.1 (7.8-14.4)	61.1 (55.2-67.0)	20.1 (15.4-24.8)	30.9 (25.4-36.4)	2.4 (0.8-4.0)	19.7 (14.6-24.8)
	Hispanic	66.2 (59.1-73.3)	17.4 (11.1-23.7)	40.1 (32.3-47.9)	29.7 (22.4-37.0)	39.5 (31.3-47.7)	4.1 (0.6-7.6)	20.0 (13.1-26.9)
	Other	65.5 (60.8-70.2)	8.4 (5.5-11.3)	45.1 (39.2-51.0)	19.7 (15.0-24.4)	40.3 (34.6-46.0)	2.7 (0.3-5.1)	18.0 (13.7-22.3)
	Multi-racial	71.6 (62.4-80.8)	18.7 (8.9-28.5)	46.6 (36.4-56.8)	29.7 (19.5-39.9)	49.1 (39.9-59.3)	6.8 (0.0-15.4)	26.8 (16.0-37.6)
**Smoking Status**	Never	69.2 (67.0-71.4)	12.1 (10.5-13.7)	47.5 (44.8-50.2)	18.6 (16.4-20.8)	34.4 (31.9-36.9)	2.6 (1.6-3.6)	19.2 (17.0-21.4)
	LT* former	78.7 (76.9-80.5)	11.5 (9.9-13.1)	52.8 (50.3-55.3)	19.2 (17.2-21.2)	34.4 (32.0-36.8)	2.2 (1.4-3.0)	17.3 (15.5-19.1)
	RT^†^ former	85.9 (81.4-90.4)	12.1 (7.6-16.6)	40.8 (34.1-47.5)	27.8 (21.5-34.1)	49.2 (42.3-56.1)	3.9 (1.0-6.8)	14.5 (9.8-19.2)
	Current	88.1 (85.9-90.3)	13.1 (10.9-15.3)	45.1 (42.0-48.2)	26.1 (23.4-28.8)	49.0 (45.9-52.1)	3.4 (2.2-4.6)	16.4 (14.2-18.6)

* Long-term: Ever smokers who quit more than a year ago at the time of survey.

^†^ Recent-term: Ever smokers who quit within a year or less at the time of survey.

Table 5 also shows that those who have heard about e-cigarettes are most likely to report television as their source of information, 48.0%. The second most likely source is “in-person conversation”, 38.2%, followed by Internet, 20.7%, and radio, 12.2% and social networks, 2.7%. 

There are some interesting differences in Table 5. For example, men are more likely to have heard about e-cigarettes than women, in general. Older people are more likely than younger people to have heard about e-cigarettes from television, while being less likely to have heard about them from the Internet. The same pattern appears in the lower and higher education groups. Smokers and recent former smokers are more likely to have heard about e-cigarettes “in-person” than long-term former smokers or never smokers, while the latter groups are more likely to have heard about them from television. There is a clear trend that television and in-person are the most common sources of awareness for e-cigarettes.

Survey respondents can report more than one source of awareness of e-cigarettes. However most, 72.1%, reported only one source when answering the survey. Another 18.4% checked off two sources. The rest, 9.5%, reported three or more sources (data not shown in Table 5).

Table 6 presents the percentage of population who can be considered susceptible to future e-cigarette use. The “susceptible” category includes all those who had ever tried e-cigarettes but were not currently using, and those who have not experimented with them but are “very likely”, or “somewhat likely” to use them in the future. The proportion of respondents susceptible to future use is dramatically different across smoking status. About 2.6% of never smokers are susceptible, but nearly half, 49.5%, of current smokers are susceptible to future use of e-cigarettes. Among those who are susceptible, an average of 56.4% have tried e-cigarettes but are not currently using them, 9.5% said they are “very likely” to use them, and 34.1 % said “somewhat likely.” 

**Table 6:** Susceptibility to Using E-Cigarettes in the Future*.

	**Never smokers (n=3,251)**	**Long-term former smokers^†^ (n=3,256)**	**Recent former smokers^‡^ (n=385)**	**Current smokers (n=2,882)**	**Overall sample (n=9,774)**
	**% (95% CI)**	**% (95% CI)**	**% (95% CI)**	**% (95% CI)**	**% (95% CI)**
**Not susceptible**	97.4 (96.6-98.2)	96.7 (95.9-97.5)	75.1 (69.6-80.6)	50.5 (47.4-53.6)	88.0 (87.1-88.9)
**All Susceptible**	2.6 (1.8-3.4)	3.3 (2.5-4.1)	24.9 (19.4-30.4)	49.5 (46.4-52.6)	12.0 (11.1-12.9)
**All Susceptible by category**					
Tried e-cigarettes	38.7 (25.4-52.0)	67.1 (56.1-78.1)	88.2 (80.7-95.6)	55.8 (51.5-60.1)	56.4 (52.6-60.2)
Very likely	14.9 (7.3-22.5)	16.0 (6.8-25.2)	1.0 (0.0-2.4)	8.8 (6.6-11.0)	9.5 (7.5-11.5)
Somewhat likely	46.4 (32.7-60.1)	16.9 (9.1-24.7)	10.8 (3.9-17.7)	35.4 (31.3-39.5)	34.1 (30.5-37.7)

* Sample excludes those who reported current use of e-cigarettes.

^†^ Smokers who quit more than a year ago at the time of survey.

^‡^ Smokers who quit within a year or less at the time of survey.

## Discussion

This study, based on a national probability sample, found that three quarters of the U.S. adult population have heard about e-cigarettes, and approximately 8% of them have experimented with e-cigarettes. Among current smokers, over 30% have ever used e-cigarettes. Of those who have ever tried e-cigarettes, about 18% are currently using them, the same transition rate as for snus use. However, both the ever use and the current use rates are about twice those for snus. Moreover, those who have tried e-cigarette are twice as likely to report using e-cigarettes as a quitting aid than snus users are to report using snus as a quitting aid. E-cigarette users are also significantly more likely to consider e-cigarettes safer than conventional cigarettes than snus users are to consider the same about snus. Finally, about half of current smokers appear to be susceptible to e-cigarette use in the future.

That 75% of U.S. population reported being aware of e-cigarettes is somewhat surprising, given that the survey was conducted before the onset of any major paid media promotion by large tobacco companies. E-cigarettes are presumably mostly of interest to current smokers, or about 20% of the U.S. population [50]. This survey found that almost 90% of current smokers have heard about them, but even two-thirds of never smokers reported having heard of e-cigarettes. When asked about where they have heard about these products, television tops the list. Internet, which we suspected to be a major driver for the spread of information about e-cigarettes [12,54], ranks third on the list. 

Since there is little data indicating major television advertising paid for by e-cigarette manufacturers before this survey was conducted (in February-March, 2012), the high level of awareness attributed to television suggests that the products might have garnered considerable earned media attention. Earned media includes national news programs or health programs that discuss the pros and cons of e-cigarettes [55-58]. It also includes celebrity endorsements on popular TV talk shows [34,35]. And it could include many local television and radio programs, which feed from these national news programs [59-61]. There is, however, no formal documentation of exactly how often this took place, and survey respondents might have based their report on what was most salient in their memory, not what was most frequent.

One reason for the media’s interest in e-cigarettes may be a novelty effect as they are relatively new products in the U.S. market. Another reason that e-cigarettes may have attracted earned media is the intuitive appeal of the products: e-cigarettes mimic regular cigarettes in so many ways that it seems to be, simply put, a clever invention. For example, as we were preparing the first draft of this paper, articles about e-cigarettes appeared in two highly regarded American publications: National Geographic and Consumer Reports [62,63]. Neither article directly promoted e-cigarettes, but both clearly stoked interest in the products. Both articles included appealing pictures of e-cigarettes. The fact that “in-person conversation” was the second most frequently reported source of information (ahead of “Internet”) in the present study also suggests that many people find the products interesting enough to raise the topic with friends and colleagues. 

A limitation of the present study is that members of the KnowledgePanel sample may engage in more than one survey in a given year, which might lead to greater familiarity with certain topics. It is possible that the rate of self-reported awareness of e-cigarettes might have been inflated for that reason, particularly because the survey described e-cigarettes before asking if respondents had heard about them. However, high rates of awareness have also been reported in other surveys. For example, a study in England found that 62% and 79% of smokers were aware of e-cigarettes in 2010 and 2012, respectively [5]. Consumer surveys in the U.S. also showed high awareness rate: In 2010 and 2011, awareness of e-cigarettes was 41% and 58%, respectively [37]. Although the consumer survey in the U.S. was not based on a probability sample of the entire population, it did show that awareness increased significantly. With these consumer survey data as a reference, the high level of awareness found in the present study suggests that interest in e-cigarettes in the U.S. continued to grow after 2011, to 75% in 2012 among the U.S population as a whole (Table 5). 

This increase in awareness is supported by the increase in the rate of ever use of e-cigarettes. The consumer survey study, referenced above, reported that 3.3% of respondents in a web-based survey had ever used e-cigarettes in 2010, which increased to 6.2% in 2011 [37]. Another smaller but population-based survey in 2010 reported an ever use rate of 1.8% [64]. The present study, which was population-based and conducted in March 2012, found 8.1% reported ever having used e-cigarettes (Figure 1), 3 to 4.5 times higher the rates found in 2010. In contrast, ever use of snus has not increased from 2010 to 2012. The ever use rates for snus from the two 2010 surveys cited were 5.4% and 5.1%, respectively [64,65]. The present study found 4.3% of respondents have ever used snus. In other words, the ever use rate for snus was at least twice as high as that of e-cigarettes in 2010. By 2012, the rate of ever use for e-cigarettes has jumped to be twice as high as that of snus, and the rate of snus use has remained essentially unchanged. 

Interestingly, this survey, based on a probability sample of the U.S. population, found that most current users of e-cigarettes use them on a non-daily basis. This differs from previous studies that recruited subjects through websites, whose samples are less likely to be representative of all e-cigarette users [14,66,67]. For example, one online survey of e-cigarette users found 81% of them were daily users [7]. The present study did find, however, that e-cigarette users who are recent former smokers are much more likely to be daily users than those who are still smoking regular cigarettes (Table 5). This could be an indication that some of these recent quitters are using e-cigarettes daily as a replacement for regular cigarettes.

Over 50% of those who have ever used e-cigarettes reported trying to quit regular cigarettes as one reason they used e-cigarettes. This is supported by data that current users of e-cigarettes are indeed more likely than non-users to have made an attempt to quit regular cigarettes in the last 12 months preceding the survey. A smaller proportion of ever users of snus reported trying to quit regular cigarettes. Like e-cigarette users, current users of snus are more likely than non-users to have made a quit attempt in the last 12 months. 

The proportion of e-cigarette users who believe that e-cigarettes are safer than regular cigarettes is significantly higher than snus users who believer snus is safer than cigarettes (50% vs. 11%). This perception may be incorrect, but it may have contributed to the large increase in experimentation with e-cigarettes from 2010 to 2012, while snus use has remained relatively constant.

The contrast in American smokers’ interest in e-cigarettes and snus is instructive in many ways. Snus has been an established and popular tobacco product in Sweden for many decades. It came to the U.S. market six years before e-cigarettes [68]. It has the support of large U.S. tobacco companies [27]. E-cigarettes, on the other hand, were first developed in China in 2003 and came to the U.S. market in 2007 [4]. Prior to the recent acquisition of the Blu e-cigarette company by Lorillard, e-cigarettes were promoted mainly by small producers. Yet, the use of e-cigarette products has grown from half that of snus in 2010 to twice that of snus by 2012. 

One reason that more smokers are experimenting with e-cigarettes than with snus, however, appears to be the following: e-cigarettes appeal to both men and women while snus appeals mainly to men. In fact, e-cigarettes appeal to women more than men (Table 2). It is possible e-cigarettes are perceived as clean nicotine devices, which might appeal to women more than men. The design and packaging of e-cigarettes and e-cigarette promotion that is specifically targeted to women may also have contributed to this gender difference [69]. In any case, the fact that more women than men have tried e-cigarettes deserves careful investigation. It is especially interesting since men are more likely to have heard about e-cigarettes than women (Table 5). No other so-called potentially reduced exposure product (PREP) has attracted more women than men.

The most striking contrast, perhaps, is between the adoption of e-cigarettes and the adoption of another product that is very similar to e-cigarettes. Premier, later called Eclipse, is almost exactly the same as an e-cigarette. It does not involve combustion when smoked [70,71], it looks like a regular cigarette, and it lights up when smoked. However, it still uses tobacco leaves. It heats the tobacco leaves to deliver nicotine to smokers. The product is reported to have cost the R.J. Reynolds tobacco company about $1 billion U.S. dollars to develop and market test [72]. There was much discussion and promotion when the product first came to market [71,73], but it never quite took off [73]. In contrast, e-cigarettes appear to have tapped into the popular imagination quickly, initially without the backing of any major tobacco company.

This study shows that nearly half of current adult smokers in the U.S. are susceptible to future use of e-cigarettes, and about 25% of the recent former smokers are susceptible. In addition, 3.3% of long term former smokers and even 2.6% of adult never smokers are susceptible. While the rates for these latter two groups are low, the size of these two groups is about 80% of the adult U.S. population. It is not clear what proportion of youth is susceptible to e-cigarette use. But the number of potential e-cigarette users among adults is already very large. All together, the rates in Table 6 translate to 29 million adults in the U.S. susceptible to e-cigarette use.

The popularity of e-cigarettes, if it continues to grow, creates a dilemma for the public health community. On the one hand, e-cigarettes are a new kind of tobacco-based product that is completely unregulated. There are numerous brands currently on market, easily purchased over the Internet or even in gas stations and convenience stores [74]. The ingredients of most brands are not reported. Safety data are lacking. Their efficacy for helping smokers to quit regular cigarettes is not well established. Their potential negative impact on tobacco control norms is unknown, especially their potential to induce adolescent nonsmokers to take up tobacco-based products. Meanwhile, many smokers believe e-cigarettes are safer than regular cigarettes. Many have used them with the hope that they would help them quit smoking regular cigarettes. A substantial proportion of smokers also find e-cigarettes cheaper than regular cigarettes (Table 4), which can contribute to the popularity of the former. All of these data suggest that smokers in the U.S. are not waiting for a consensus view from health authorities to decide if they should switch to e-cigarettes. The results of the present study and those of previous studies suggest that e-cigarettes are likely to gain users in the next few years regardless of the opinions of the scientific community. 

The fact that e-cigarettes have quickly surpassed snus in perceptions related to safety and utility, and in actual use among U.S. smokers suggests that some feature of e-cigarettes must have tapped into smokers’ intuitive preferences. Whether these beliefs are correct or not, they could potentially be channeled into a productive public health campaign to increase the rate of current smokers trying to quit cigarettes. Given that the population smoking cessation rate has not improved in the last twenty years in the U.S., any measure that could increase the rate of smokers attempting to quit deserves consideration [75]. The rate of current use of e-cigarettes is still relatively low, and there has been no study suggesting that their coming to the market has led to any detectable change in the quit attempt rate at the population level. But more research on e-cigarettes or similar products that have a strong intuitive appeal may help in developing a conceptual model and corresponding policy to increase the population cessation rate. 

The case of e-cigarettes and their rapid adoption, in conjunction with the lack of scientific data on safety and efficacy, presents a difficult regulatory problem. It is imperative that the scientific community rise to the challenge. The usual approach to research for any product intended to help smokers quit using regular cigarettes proceeds from safety to efficacy. Such a process usually takes many years, and millions may be using e-cigarettes before that process is completed. Studies are needed to assess risks and benefits of these new products for individual users more rapidly. Equally important, studies are needed to identify factors that influence the population use patterns and to determine how individual preference for various products translates into benefit or harm on the population level. 
